# Single-Cell RNA Sequencing Revealed a 3-Gene Panel Predicted the Diagnosis and Prognosis of Thyroid Papillary Carcinoma and Associated With Tumor Immune Microenvironment

**DOI:** 10.3389/fonc.2022.862313

**Published:** 2022-03-11

**Authors:** Zuoyu Chen, Yizeng Wang, Dongyang Li, Yuting Le, Yue Han, Lanning Jia, Caigu Yan, Zhigang Tian, Wenbin Song, Fuxin Li, Ke Zhao, Xianghui He

**Affiliations:** ^1^ Department of General Surgery, Tianjin Medical University General Hospital, Tianjin, China; ^2^ Department of General Surgery, The People’s Hospital of Liuyang, Changsha, China

**Keywords:** papillary thyroid carcinoma, single-cell RNA sequencing, differential expression analysis, multi-omics, tumor immune microenvironment, diagnosis, prognosis

## Abstract

**Objective:**

The objective of this research was to screen prognostic related genes of thyroid papillary carcinoma (PTC) by single-cell RNA sequencing (scRNA-seq), to construct the diagnostic and prognostic models based on The Cancer Genome Atlas Thyroid Cancer (TCGA-THCA) data, and to evaluate the association between tumor immune microenvironment and the prognostic model.

**Method:**

The differentially expressed genes (DEGs) and tumor evolution were analyzed by scRNA-seq based on public databases. The potential regulatory networks of DEGs related to prognosis were analyzed by multi-omics data in the THCA. Logistic regression and Cox proportional hazards regression were utilized to construct the diagnosis and prognostic model of PTC. The performance of the diagnostic model was verified by bulk RNA sequencing (RNA-seq) of our cohort. The tumor immune microenvironment associated with the prognostic model was evaluated using multi-omics data. In addition, qRT-PCR was performed on tumor tissues and adjacent normal tissues of 20 patients to verify the expression levels of DEGs.

**Results:**

The DEGs screened by scRNA-seq can distinguish between tumor and healthy samples. DEGs play different roles in the evolution from normal epithelial cells to malignant cells. Three DEGs ((*FN1*, *CLU*, and *ANXA1*)) related to prognosis were filtered, which may be regulated by DNA methylation, RNA methylation (m6A) and upstream transcription factors. The area under curve (AUC) of the diagnostic model based on 3-gene in the validation of our RNA-seq was 1. In the prognostic model based on 3-gene, the overall survival (OS) of high-risk patients was shorter. Combined with the clinical information of patients, a nomogram was constructed by using tumor size (pT) and risk score to quantify the prognostic risk. The age and tumor size of high-risk patients in the prognostic model were greater. In addition, the increase of tumor mutation burden (TMB) and diversity of T cell receptor (TCR), and the decrease of CD8^+^ T cells in high-risk group suggest the existence of immunosuppressive microenvironment.

**Conclusion:**

We applied the scRNA-seq pipeline to focus on epithelial cells in PTC, simulated the process of tumor evolution, and revealed a prognostic prediction model based on 3 genes, which is related to tumor immune microenvironment.

## Introduction

Thyroid papillary carcinoma (PTC) is the most increased endocrine malignant tumor in recent years (1). The incidence of PTC accounts for 90% of all thyroid cancers, of which 60% are less than 2 cm, however, the mortality has not increased significantly (2). This is also one of the bases of active surveillance (AS) (3). In addition, the possibility of a real increase in the disease still exists, as well as poor survival in the advanced tumor stage, even with appropriate treatment (4). Therefore, accurate prediction of individual prognostic risk is of great significance for patient management. The American Joint Committee on Cancer (AJCC), the American Thyroid Association (ATA), and the European Thyroid Association (ETA) are now the most widely utilized risk classification systems (5–7). These traditional risk stratification systems are widely applied to predict the overall prognosis of patients. However, molecular genetics are not incorporated into these systems, on which precision evaluation and target therapy are dependent.

Over the past decade, RNA sequencing (RNA-seq) has become an indispensable tool for transcriptome-wide analysis of differential gene expression (8). At present, significant progress has been made in the analysis of prognostic related genes, and it is possible to predict the prognosis more accurately at the molecular level (9). Molecular alterations revealed by gene microarray and bulk RNA sequencing (RNA-seq) have been widely used for tumor diagnosis and prognosis prediction. However, to obtain raw and authentic tumor cell genetic information, an analysis of the transcriptome performed at the single-cell level is more valuable. Single-cell RNA sequencing (scRNA-seq) overcomes the limitations of RNA-seq—for example, using RNA-seq, only the average expression of genes is obtained, and it is difficult to study heterogeneous systems—making it possible to conduct more detailed profiling of tumor cells and their molecular changes (10). Single-cell RNA sequencing enables us to penetrate the tumor microenvironment based on cell-specific changes in the transcriptome, and further develop diagnostic and prognostic markers to aid in the precise diagnosis and treatment of patients.

In this study, we extracted scRNA-seq data from the Gene Expression Omnibus (GEO) database to analyze DEGs between tumor cells and normal epithelial cells, the trajectory and enriched signaling pathways of these genes in tumor progression were analyzed. Transcriptome data from THCA were combined to obtain 3 DEGs associated with prognostic and validated in our cohort. The potential regulatory networks of 3DEGs were analyzed in multiple dimensions. The 3 DEGs based diagnostic model of PTC was constructed by logistic regression and validated by our cohort. Meanwhile, a prognostic risk prediction model was also built using this 3-gene panel. To simplify the evaluation steps, we performed nomograms to quantify prognostic-related indicators and predict 1-, 5-, and 10-year overall survival of patients. Tumor mutational burden (TMB) and TCR diversity were also investigated to explore the underlining mechanism of risk stratification.

## Materials and Methods

### Data Collection, Processing and Downstream Analysis

Single cell RNA sequencing data are extracted from the Gene Expression Omnibus (GEO) database (GSE184362) (11). The downstream analysis of scRNA-seq (namely, quality control, dimensionality reduction, clustering, differential expression analysis, etc.) is carried out according to the standard process of Seurat (12). We applied the Wilcoxon rank sum test to determine the DEGs between the two groups of cells. Monocle2 package was used for pseudotime analysis and visualization (13). The mRNA expression data of the TCGA-THCA and GETx was downloaded from the UCSC XENA, which is converted to TPM format and standardized with log2 (14). The mutation information of the TCGA-THCA was obtained from the Genomic Data Commons (GDC). TMB was analyzed by “maftools” package (15). The list of DEGs and its prognostic information from bulk RNA-seq were queried from the GEPIA2 database (16). The principal component analysis (PCA) in the GEPIA2 was applied to reduce the dimension of the samples from the THCA and GETx.

### Protein–Protein Interaction (PPI) and GSEA Enrichment Analysis

We employed two common protein interaction databases (17, 18), the STRING and the GENEMANIA, to analyze the protein interaction and related functions of DEGs. All DEGs were sorted according to averagelog2FC, and then GSEA enrichment analysis was carried out by using ‘clusterprofiler’ with MSigDB as reference (19, 20). The results were screened according to P. adjust <0.05.

### Clinical Samples and Ethical Statements

Bulk RNA-seq was performed on 10 paired PTC tumor tissues corresponding to adjacent non-tumor tissues from the General Hospital of Tianjin Medical University. RT-PCR was performed on tumor and adjacent normal samples from another 20 paired patients. The study protocol was approved by the ethics committee of the General Hospital of Tianjin Medical University (IRB2021-KY-022). All experiments were conducted in accordance with relevant regulations, and all patients provided written informed consent.

### Quantitative Real-Time Polymerase Chain Reaction (qRT-PCR) and Immunohistochemistry

TRIzol lysate isolated total mRNA from tissues. Total RNA was obtained using the RNeasy Mini kit (Vazyme, China), and 1 μg of RNA was reverse transcribed using the PrimeScript RT reagent Kit with gDNA Eraser (Vazyme, China). Power SYBR green PCR master mix was for qRT-PCR. The −2ΔΔCt method was applied to determine relative quantification. The amount of GAPDH mRNA was chosen to standardize the relative expression of messenger RNA (mRNA) for each gene. The primer pairs were:

*FN1* forward primer, 5’-CGGTGGCTGTCAGTCAAAG-3’ and reverse primer, 5’-AAACCTCGGCTTCCTCCATAA-3’.
*CLU* forward primer, 5’-CTACTTCTGGATGAATGGTGACC-3’ and reverse primer, 5’-CGGGTGAAGAACCTGTCCT-3’.
*ANXA1* forward primer, 5’-CTAAGCGAAACAATGCACAGC-3’ and reverse primer, 5’-CCTCCTCAAGGTGACCTGTAA-3’
*GAPDH* forward primer, 5’-AGGGCTGCTTTTAACTCTGGT-3’ and reverse primer, 5’-CCCCACTTGATTTTGGAGGGA-3’.

The Human Protein Atlas (THPA, version 21) is a database of immunohistochemistry (IHC) data which can investigate protein expression in human tissues and cells (21).

### Evaluation of Gene Regulatory Networks Using Multi-Omics Databases

The regulatory network of three prognostic related DEGs were analyzed by multi-omics data. CBioportal can quickly obtain the correlation between molecular spectrum and clinical prognostic of large-scale cancer genomics projects (22). The DNMIVD database can simultaneously provide CpG based diagnostic and prognostic models (23). The database comprehensively provides 14 TCGA and 23 TCGA cancer diagnosis and prognostic models based on DNA methylation. The relationship between DNA methylation sites and gene expression levels was evaluated by MEXPRESS (24). ChEA3 is a transcription factor (TF) enrichment analysis tool (25). The background database contains a gene library generated from multiple sources, namely, TF gene co-expression from RNA-seq study, TF target association from Chip-seq experiment, and TF gene co-expression calculation from the gene list submitted by the population. RMVar contains 9 RNA modifications, namely, m6A, m6Am, m1A, pseudouridine, m5C, m5U, 2’-O-Me, A-to-I, and m7G (26). We calculated the correlation between DEGs and m6A related genes (27, 28).

### Construction of Diagnostic Model and Prognostic Model

Both Kaplan–Meier (KM) analyses and log-rank tests were employed to assess overall survival (OS) and progression-free survival (PFS) of 16 hub DEGs. The diagnostic model of THCA was built by logistic regression (‘glm’ function in R), score = –2.760 + (0.713 ∗ CLU) + (0.018 ∗ ANXA1) + (–0.987 ∗ FN1), cut-off was –2.628. The Hosmer–Lemeshow test was applied to measure the fitting quality of diagnostic model in THCA data and our cohort. The prognostic model of THCA was built by Cox proportional hazards regression (‘survival’ R package), score = 3.705 + (–0.089 ∗ CLU) + (–0.448 ∗ ANXA1) + (0.151 ∗ FN1). The integrated nomogram incorporated tumor size and risk score. To examine various survival determinants, we created time-dependent receiver operating characteristic (ROC) curves. The “pROC” R package calculated the area under the curve (AUC). R package ‘rms’ constructed the nomogram. The performance of the nomograms was evaluated by calibration.

### Evaluation of Immune Infiltration and Immune Score

We acquire more confidence in the findings seen by multiple approaches since TIMER2.0 (29) employs six alternative calculation methods to evaluate immune cell infiltration in THCA tumors or transcriptome data given by users (30). The Pan-Cancer Atlas initiative compares the 33 tumor types described by the TCGA. We obtained the relevant immune score and immune repertoire information in the TCGA-THCA (31). The ‘ESTIMATE’ algorithm calculates the immune-stromal component ratio in tumor microenvironments (32).

### Statistical Analysis

For comparisons between the two groups, Student’s t-tests were facilitated to ascertain statistical significance for normally distributed variables, while Mann–Whitney U tests were used for nonnormally distributed data. Statistical significance was described as follows: ns, not significant; *, p <0.05; **, p ≤0.01; ***, p ≤0.001.

## Results

### Single-Cell RNA Sequencing Reveals Cell Distribution and DEGs in Tumor Cells and Normal Cells

**Figure 1:**
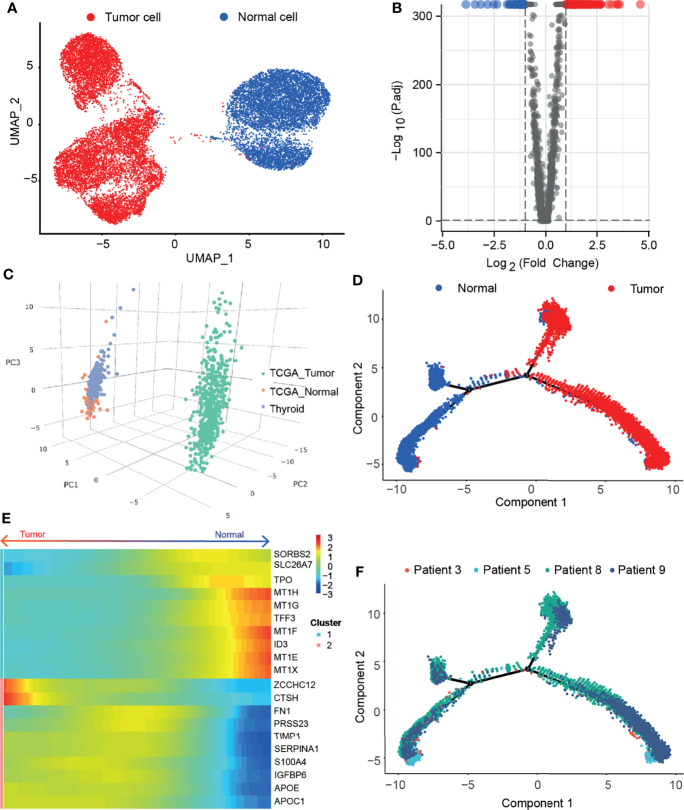
Single cell sequencing reveals differentially expressed genes in thyroid papillary carcinoma. **(A)** Umap of tumor cells and adjacent normal cells in paired samples of 4 PTC patients. **(B)** Volcano map of DEGs. **(C)** PCA of 130 DEGs in THCA and GETx. **(D)** Pseudotime analysis of malignant transformation of normal cells. **(E)** Heatmap of the 10 most significantly upregulated and downregulated genes among the DEGs. **(F)** Patient information in the transformation trajectory from normal cells to malignant cells.

We obtained scRNA-seq expression matrices of 126,646 cells from tumor tissues and adjacent normal tissues of 4 paired PTC patients from the GEO database. After quality control, 11,390 tumor cells (*TG*, *EPCAM*, *KRT18*, and *KRT19*) and 6,808 normal cells (*TG*, *TPO*) were obtained for subsequent analysis (**Supplementary Figures 1A–D**). Then we performed the nonlinear dimensionality reduction method uniform manifold approximation and projection (UMAP) to reduce the dimension of cells (**Figure 1A**). Taking |averageLog2FC| >1 and adj. P < 0.05 as the threshold, a total of 130 DEGs (86 upregulated and 44 downregulated) between tumor cells and normal cells were obtained and displayed in a volcano plot (**Figure 1B**). After uploading the DEGs to the GEPIA2 (16), these genes can distinguish tumor tissue from normal tissue (**Figure 1C**). We applied monocle2 to perform pseudotime analysis (13) and found that they have potential transformation trajectories from normal cells to malignant cells (**Figures 1D, F**). In addition, we also demonstrated the 10 most significantly upregulated and downregulated genes among the DEGs (**Figure 1E**). It is suggested that DEGs may have different expression patterns during the transformation of normal cells to malignant cells.

### Protein Interaction Network and GSEA Enrichment Analysis of DEGs

**Figure 2:**
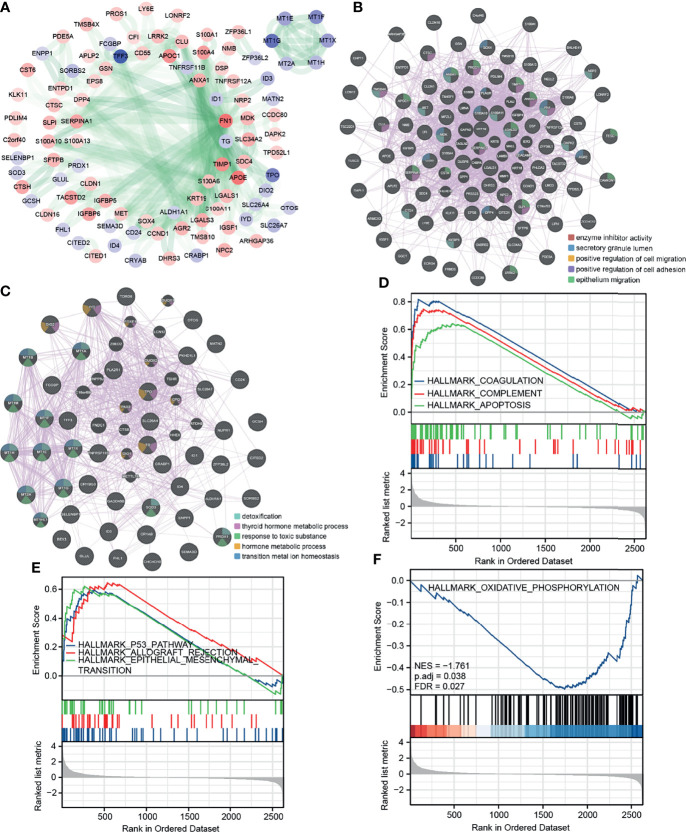
Protein interaction network and GSEA enrichment analysis of DEGs. **(A)** PPI of the DEGs in STRING database. **(B, C)** PPI and functional enrichment analysis for upregulated and downregulated DEGs in GENEMANIA database, respectively. **(D, E)** GSEA analysis HALLMARK pathway (upregulated in tumor cells). **(F)** GSEA analysis HALLMARK pathway (upregulated in normal cells).

We predicted the protein–protein interaction (PPI) of the DEGs in the STRING database (**Figure 2A**). The node color was assigned according to the fold change, and the transparency of the line represented the strength of the interaction between the two proteins. Then, for upregulated and downregulated DEGs, we utilize the GENEMANIA database to determine protein–protein interactions and a few functional enrichment analyses (**Figures 2B, C**). We found that upregulated genes were enriched for enzyme inhibitory activity, secretory granules, and cell migration; downregulated genes were enriched for detoxification and thyroid hormone synthesis. We applied a GSEA analysis to assess the distribution trends of genes in a predefined gene set in a gene table ranked by their relevance to phenotype, to determine their relationship to HALLMARK-related pathways (**Figures 2D–F**). Pathways significantly enriched in tumor cells were: coagulation, complement, apoptosis, p53 pathway, allograft rejection, and epithelial–mesenchymal transition (EMT). A pathway significantly enriched in normal cells was oxidative phosphorylation.

### Screen Hub DEGs and Explore the Expression of 3 Prognosis-Related Genes

**Figure 3:**
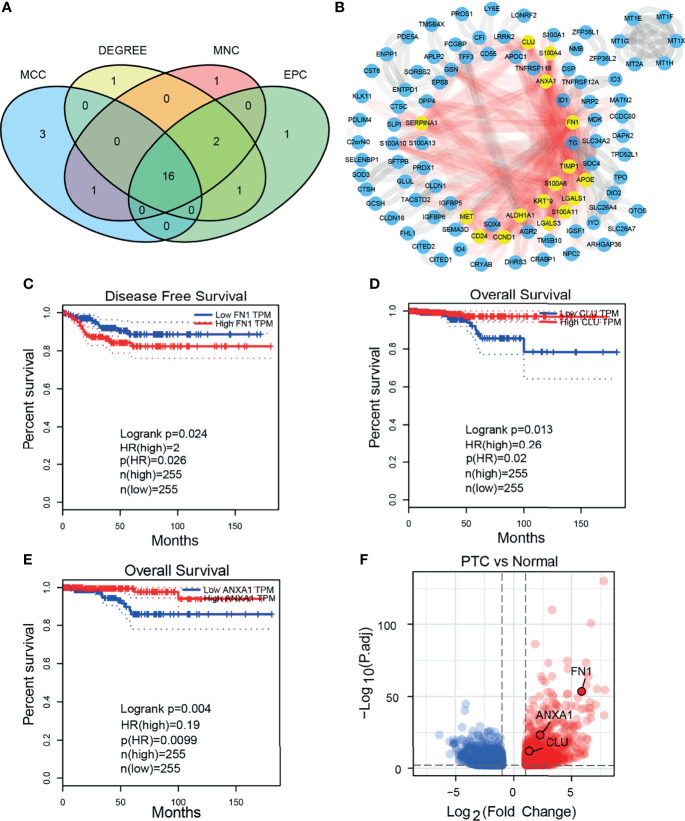
Hub DEGs and 3 prognostic related DEGs. **(A)** Venn plot of 16 hub DEGs screened by four algorithms in CytoHubba. **(B)** 16 hub DEGs in PPI network. **(C–E)** The 3 prognostic related DEGs analyzed by GEPIA2 (both Disease Free Survival and Overall Survival). **(F)** GSEA analysis HALLMARK pathway (upregulated in normal cells).

**Figure 4:**
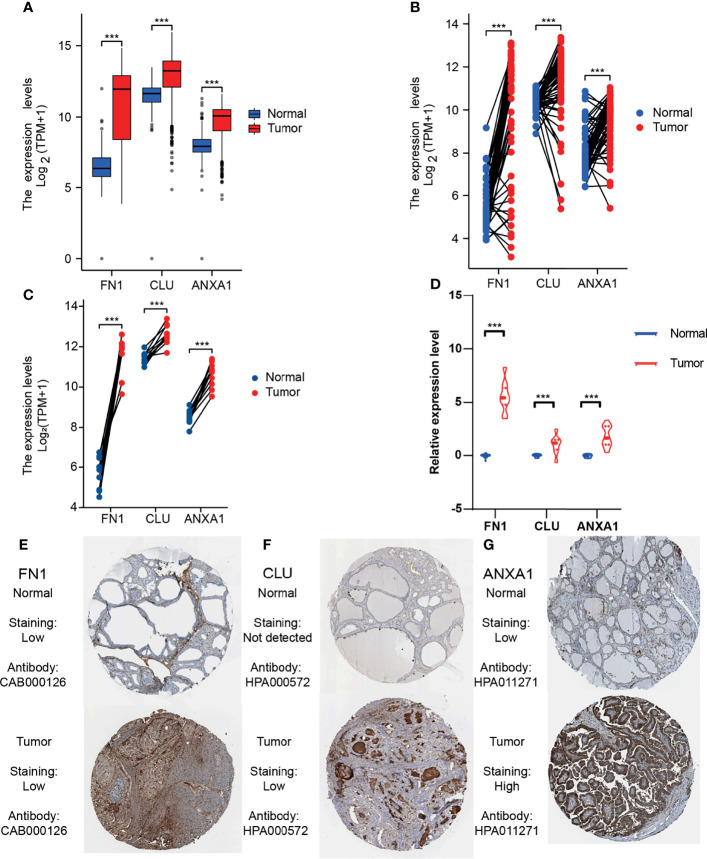
Expression level of 3 prognostic related DEGs in THCA and our cohort. **(A)** Expression level of 3 DEGs in unpaired THCA samples. **(B)** Expression level of 3 DEGs in 58 paired THCA samples. **(C)** Expression level of 3 DEGs in our 10 paired bulk RNA-seq. **(D)** Relative expression level of 3 DEGs in our 20 paired samples (qRT-PCR). **(E–G)** The immunohistochemical staining degree of 3 DEGs in the THPA (Antibodies: CAB000126, HPA000572, and HPA011271). ***p≤0.001.

We obtained 16 hub DEGs (**Figure 3A**) using 4 algorithms (33) in CytoHubba and displayed in the PPI network (**Figure 3B**), of which 14 were upregulated and 2 were downregulated. Three of these genes (*FN1*, *CLU*, and *ANXA1*) were found to be associated with prognosis in the GEPIA2 database (**Figures 3C–E**), with Logrank P-value 0.024, 0.013, and 0.004, respectively. The expression of 3 prognostic related genes was validated by bulk RNA-seq from our cohort (**Figure 3F**). Using bulk RNA-seq results from 850 samples from the THCA and GETx, 58 paired samples, 10 paired samples from our cohort and paired qPCR results of 20 patients, we verified that 3 DEGs were all overexpressed in tumor tissues (**Figures 4A–D**). Then, the protein expression levels of the 3 hub genes were verified in the THPA (**Figures 4E–G**). In the cell subsets of tumor samples (**Supplementary Figure 1A**), FN1, CLU, and ANXA1 were high expressed in tumor cells and low expressed in myeloid cell and fibroblast. In the cell subsets of normal samples (**Supplementary Figure 1C**), FN1 and ANXA1 were high expressed in tumor cells and low expressed in myeloid cell and fibroblast; CLU was low expressed in tumor cells and high expressed in myeloid cell and fibroblast. In paired samples (**Figure 4B**), there is disturbance in the expression of 3 prognostic related genes, suggesting that individual gene may not be able to fully predict the prognosis of tumor patients.

### Potential Regulatory Networks of Three Core Genes

**Figure 5:**
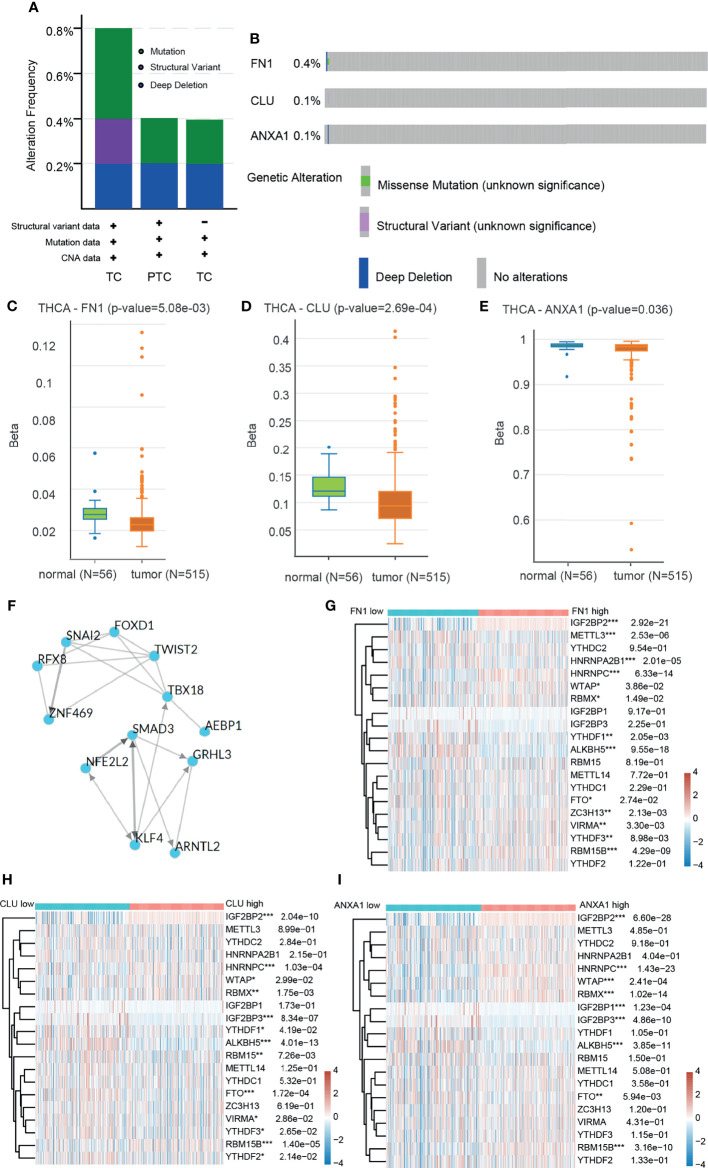
Analysis of regulatory network based on multi-omics data. **(A)** The overall frequency of 3 prognostic related genome-level alteration in THCA (Firehose Legacy), PTC (Cell 2014), THCA (PanCancer Atlas) of DEGs. **(B)** The separated frequency of 3 prognostic related genome-level alteration of DEGs in THCA (Firehose Legacy), PTC (Cell 2014), THCA (PanCancer Atlas). **(C–E)** DNA methylation levels of 3 DEGs between tumor tissues and adjacent tissues in THCA. **(F)** Interaction between transcription factors regulating 3 DEGs. **(G–I)** Heatmap of the relationship between m6A related genes and 3 DEGs. *p≤0.05; **p≤0.01; ***p≤0.001.

We explored the potential regulatory networks of 3 prognostic related DEGs in tumor samples using a multi-omics platform. We analyzed genome-level changes in the CBioportal database. In 3 datasets, the overall genomic variation for these genes was less than 0.8% (**Figure 5A**) and the genome-level variation for each gene was less than 0.4% (**Figure 5B**). It is suggested that these gene-level changes are less affected by mutations. And these genomic-level alterations were not associated with overall patient survival (**Supplementary Figure 2A**). We further analyzed the DNA methylation levels of these genes. The methylation levels of genes in tumor tissues were significantly lower than those in normal tissues (**Figures 5C–E**), suggesting that genomic methylation is a potential regulator of the high expression of 3 genes (**Supplementary Figures 2B–D**). Determining gene expression changes regulated by transcription factors (TFs) is an important step in understanding gene regulatory networks. Using ChEA3, we were able to predict a regulatory network, namely, 12 transcription factors that regulate 3 prognostic related genes (**Figure 5F**). The regulation of gene expression is multi-dimensional, and we found potential transcriptome epigenetic regulation sites (m6A regulatory sites) of these genes in the RMVar database. Therefore, the tumor tissues were divided into two groups by gene expression level from the THCA, and the changes of m6A-related genes were compared (**Figures 5G–I**). The expression of 3 DEGs was significantly negatively correlated with 2 scavenging genes (*FTO*, *ALKBH5*), positively correlated with 2 reading genes (*HNRNPC*, *IGF2BP2*), and positively correlated with 2 writing genes (*WTAP*, *RBM15B*). These results suggest that the high expression of 3 genes in tumors may be regulated by DNA methylation and mRNA methylation modification.

### The Diagnosis and Prognostic Model of PTC Was Constructed and Featured in Nomogram

**Figure 6:**
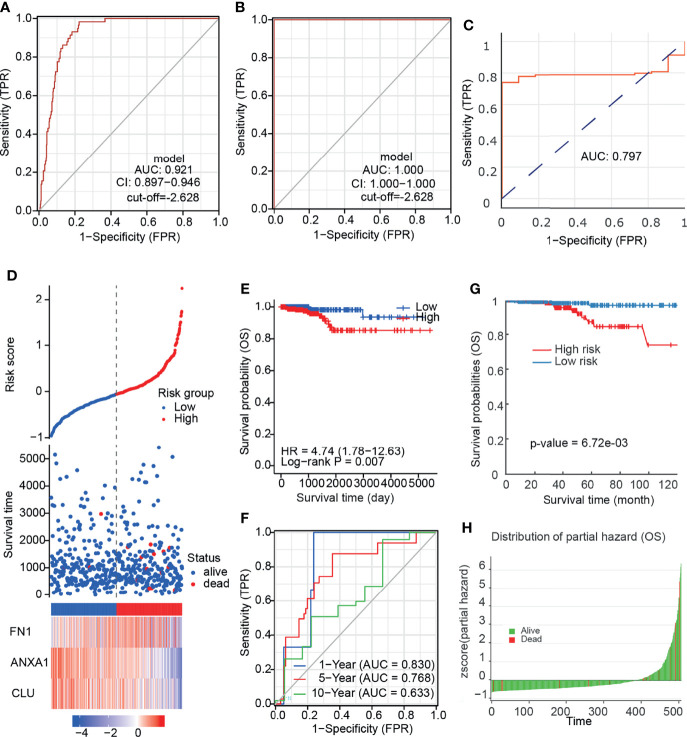
The diagnosis and prognostic model of PTC. **(A)** ROC curve of diagnostic model based on mRNA expression. **(B)** ROC curve of diagnostic model validation set based on mRNA expression. **(C)** ROC curve of diagnostic model based on DNA methylation. **(D)** Risk factor association diagram based on mRNA expression. **(E)** KM plot of prognostic model based on mRNA expression. **(F)** ROC curve predicted by prognostic model for different follow-up time. **(G)** KM plot of prognostic model based on DNA methylation. **(H)** Z-score distribution of the prognostic model and survival status.

Due to the disturbance in the expression of the 3 prognostic related DEGs in the paired samples, we speculate that a comprehensive scoring model based on these genes may be beneficial for the diagnosis and prognosis of PTC. We constructed a diagnostic model ROC curve based on mRNA expression levels, with a cutoff value of −2.628 for the score and an area under the curve (AUC) of 0.921 (**Figure 6A**). This was subsequently validated using our own cohort with AUC: 1 (**Figure 6B**). It is suggested that the diagnostic effect of this model is stable. We utilized the DNMIVD database to construct a diagnostic model based on DNA methylation levels, and the AUC was 0.797 (**Figure 6C**), suggesting that its diagnostic efficacy was moderate. We did not validate because the validation set is missing. We further constructed a prognostic prediction model by mRNA expression (**Figure 6D**) and divided patients into high and low risk groups. KM survival analysis found that patients in the high-risk group had worse prognosis (**Figure 6E**), and the model predicted 83, 76.8, and 63.3% of overall survival for patients at 1, 5, and 10 years, respectively (**Figure 6F**). It is suggested that the model prediction effect is moderate. Due to the lack of survival data on the external dataset, we failed to validate it. We also constructed a prognostic model based on DNA methylation levels using the DNMIVD database, suggesting that patients in the high-risk group had a worse prognosis (**Figures 6G, H**).

**Figure 7:**
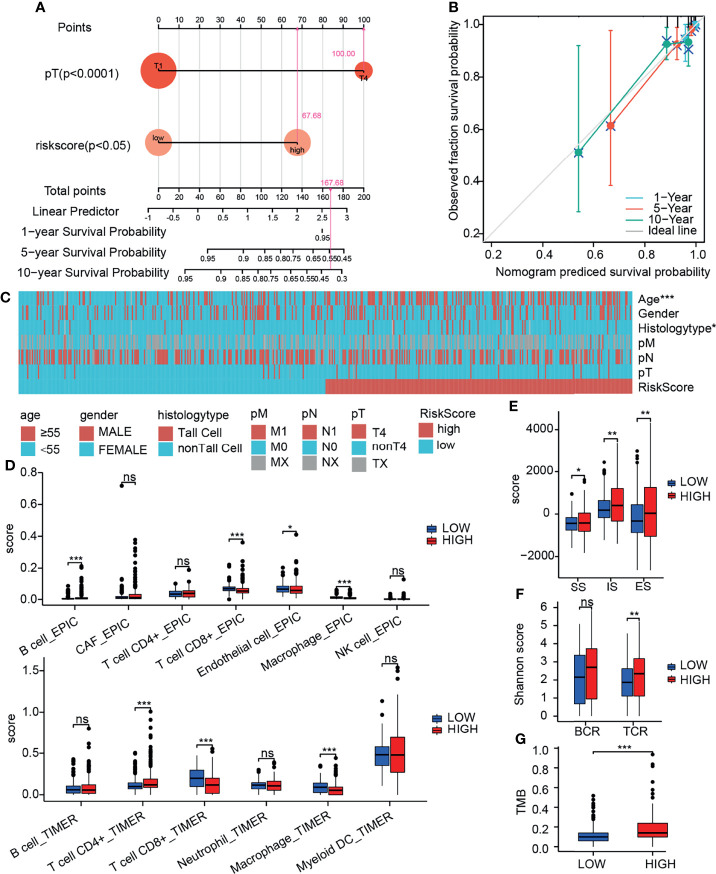
Integrative analysis of prognostic model. **(A)** Nomogram quantifies clinical information and risk score. **(B)** Calibration curve to evaluate nomogram model. **(C)** Heatmap of the relationship between prognostic risk score and clinical information. **(D)** Relationship between prognostic risk score and immune cell infiltration (by TIMER and EPIC). **(E)** Relationship between prognostic risk score and immune score (by ESTIMATE). **(F)** Relationship between prognostic risk score and TMB. **(G)** Relationship between prognostic risk score and diversity of immune repertoire. ns, not significant; *p≤0.05; **p≤0.01; ***p≤0.001.

To further quantify the prognostic risk score at the transcriptome level and the impact of clinical factors on the overall survival of patients, we constructed nomograms using Cox regression analysis (**Table 1**). In multivariate Cox regression analysis, pT4 and high-risk group were found to be independent risk factors related to prognosis, and a nomogram simplified evaluation system was constructed (**Figure 7A**). In the calibration plot, we found that the model can fit well with the diagonal (**Figure 7B**), suggesting the reliability of the model.

**Table 1:** Univariate and multivariate Cox regression analysis of clinical information and risk score.

Characteristics	Total (N)	Univariate analysis		Multivariate analysis	
		Hazard ratio (95% CI)	P-value	Hazard ratio (95% CI)	P-value
age	510				
<45	231	Reference			
≥45	279	722839341.155 (0.000–Inf)	0.997		
gender	510				
FEMALE	371	Reference			
MALE	139	1.963 (0.710–5.428)	0.193		
pM	295				
M0	286	Reference			
M1	9	4.258 (0.909–19.952)	0.066	1.967 (0.380–10.172)	0.420
pN	460				
N0	229	Reference			
N1a	90	0.888 (0.171–4.602)	0.888		
N1	59	1.358 (0.321–5.748)	0.677		
N1b	82	2.538 (0.601–10.714)	0.205		
pT	508				
T1	485	Reference			
T4	23	9.248 (3.339–25.611)	<0.001	5.034 (1.322–19.166)	0.018
Risk score	510				
Low	255	Reference			
High	255	4.744 (1.351–16.655)	0.015	10.422 (1.330–81.670)	0.026

### Integrative Analysis of Prognostic Model

We further evaluated the clinical information, TMB, immune infiltration and immune score of patients with different prognostic risk score. We found that the high-risk group was associated with higher patient age and invasive subtypes (**Figure 7C**). We discovered a substantial drop in CD8^+^ T cells and macrophages in the high-risk group using the TIMER deconvolution model and the suggested EPIC algorithm in TIMER2.0 (**Figure 7D**). The ImmuneScore (IS), StromalScore (SS), and EstimateScore (ES) make up the ESTIMATE evaluation. In the high-risk group, IS, SS, and ES were significantly increased, suggesting increased infiltration of immune cells and stromal cells (**Figure 7E**). TCR diversity was significantly increased in the high-risk group (**Figure 7F**). TMB was significantly increased in the high-risk group (**Figure 7G**). The method of gene set score further evaluated the tumor immune microenvironment in different risk groups (**Table 2**) (31). The tumor proliferation was significantly enhanced in the high-risk group. The mutation rate was significantly increased in both silent mutation rate and nonsilent mutation rate. These changes may be one of the reasons for the significant increase of SNV neoagents in the high risk group. Persistent antigen stimulation may also lead to T cell anergy or reduction. The sum of amplified or deleted (collectively “altered”) arms was used to determine aneuploidy scores. Aneuploidy score was also significantly higher in the high-risk group. The fraction altered score change in copy number burden also increased significantly in high risk group. Homologous recombination defects (HRD) score combines three measures of genomic scarring: large (>15 Mb) non-arm-level areas with LOH, large-scale state transitions (breaks between contiguous segments of >10 Mb), and subtelomeric regions with allelic imbalance to evaluate abnormalities in homologous recombination. HRD increased significantly in the high-risk group. Leukocyte fraction infers from the DNA methylation probes that the proportion of leukocytes increased significantly in the high-risk group. These results suggest that the tumor cells in the high-risk group grow rapidly, and the increase of chromosome abnormal amplification and TMB may lead to the increase of tumor heterogeneity. It is indirectly proved that these patients have poor prognosis. In addition, tumor infiltrating immune cells increased; however, the decrease of CD8 ^+^ T cells and the increase of TCR diversity imply that there is an immune escape phenotype in high risk patients. There was no significant difference in the scores of IFN gamma response and TGF beta response between the two groups.

**Table 2:** Gene set score characteristics of different prognostic risk groups.

Character	High risk	Low risk	T	P-value
	(mean ± SD)	(mean ± SD)		
Leukocyte Fraction	0.1657 ± 0.1441	0.1103 ± 0.0849	5.2238	<0.0001
Proliferation	−1.506 ± 0.4436	−1.6442 ± 0.3774	3.7407	0.0002
Wound Healing	−0.2648 ± 0.0814	−0.2739 ± 0.0788	1.2739	0.2033
Macrophage Regulation	−0.1967 ± 0.7678	−0.25 ± 0.5075	0.9132	0.3617
IFN-gamma Response	−0.3298 ± 0.6123	−0.3508 ± 0.5021	0.4167	0.6771
TGF-beta Response	−0.1127 ± 0.3948	−0.077 ± 0.2707	−1.177	0.2398
SNV Neoantigens	6.1617 ± 5.1199	4.5164 ± 3.5929	4.0572	<0.0001
Indel Neoantigens	11.4043 ± 20.6571	10.0769 ± 22.261	0.405	0.686
Silent Mutation Rate	0.1077 ± 0.1112	0.0742 ± 0.077	3.8519	<0.0001
Nonsilent Mutation Rate	0.3355 ± 0.2598	0.2332 ± 0.1556	5.2605	<0.0001
Number of Segments	51.8537 ± 14.7062	50.3902 ± 11.8516	1.2152	0.2249
Fraction Altered	0.0562 ± 0.1766	0.0112 ± 0.0608	3.7802	0.0002
Aneuploidy Score	1 ± 3.3588	0.2026 ± 1.348	3.3506	0.0009
Homologous Recombination Defects	0.6121 ± 1.6737	0.3216 ± 1.0839	2.2117	0.0276
BCR Shannon	2.4196 ± 1.5996	2.105 ± 1.5211	1.5499	0.1225
TCR Shannon	2.2245 ± 1.2885	1.8856 ± 1.121	2.9881	0.003

## Discussion

Tumor microenvironment is a complex ecosystem of malignant cells, immune cells, and stromal cells. PTC grows slowly and cervical lymph node metastasis is common, but tumor metastasis is rare (2). Some untreated tumors can dormancy or even shrink spontaneously (34), lymph node metastasis disappears spontaneously (35), but immune surveillance can fade in certain conditions (36). Tumor in advanced stage usually showed immunophenotype dominated by immune escape (4). Single cell RNA-seq offers a unique perspective on the mechanism of tumor internal and external driving responses, allowing us to focus on tumor cell alterations. As a result, the molecular prognostic prediction model developed on the basis of scRNA-seq, may effectively identify patients with high prognostic risk, complement the traditional staging prediction system, and assist individualized treatment to achieve better therapeutic effect.

We identified 130 DEGs between tumor cells and normal epithelial cells by scRNA-seq. They completely distinguish between tumor tissue and adjacent normal tissue. Pseudotime analysis showed that the expression degree of these genes was not polarized, but had the characteristics of continuous change. This reflects the heterogeneity of gene expression in tumors. We analyzed the protein–protein interaction of DEGs and conducted a GSEA enrichment analysis, suggesting that the highly expressed genes in tumor cells are related to tumor formation and progression. The highly expressed genes in normal cells are related to thyroid hormone synthesis and enriched in oxidative phosphorylation signal pathway. Because gene transcription is impulsive and there is batch effect in the experimental process (10), the DEGs we obtained are limited. In the future, to obtain more high-quality information, we need to further eliminate the batch effect and optimize the algorithm.

Combined with the THCA data, 3 high expressed DEGs (*FN1*, *CLU*, and *ANXA1*) related to the prognosis of PTC. This is consistent with our bulk RNA-seq results and staining degree of immunohistochemistry. *FN1* overexpression is an important feature of EMT and is also considered as a molecular marker to distinguish malignant lesions (37). Immunohistochemical evidence showed that the increased expression of *FN1* was mainly limited to the invasive front of thyroid cancer (38).*CLU* is a glycoprotein, which is involved in many physiological and pathological processes essential to carcinogenesis and tumor growth (39). *CLU* is an effective biomarker for more accurate evaluation of thyroid nodules (40). A previous study has preliminarily studied the role of *ANXA1* in PTC, and its high expression may be related to tumorigenesis and development in PTC (41). Based on proteomics, *ANXA1* was found to be an effective marker for the diagnosis of PTC (42).

Based on the study of these 3 DEGs in PTC, we further explored the potential networks regulating their expression. At the genomic level, we analyzed the changes at the genomic level by CBioportal and found that the overall genomic changes of the 3 genes were less than 0.8%. Compared with normal thyroid samples, targeted DNA methylation studies showed that hypomethylation rather than hypermethylation was more likely to occur in PTC tumor samples (43). We found that the DNA methylation level of these 3 DEGs decreased significantly in tumor tissues of patients, which may be a potential reason for the high expression of genes in tumor tissues. In terms of upstream regulation, we further explored the potential regulation of these three genes by 12 interacting transcription factors, but failed to explore further. Gene expression regulation is multidimensional. We found m6A potential regulatory sites in these three genes in the RNA epigenetic database (26). In 450 patients with PTC, most of the m6A RNA modification regulators were found to be downregulated, and a risk model was constructed based on 3 m6A RNA modification regulator genes (*FTO*, *RBM15*, and *KIAA1429*) that may be used as an independent predictive biomarker in PTC (44). These results suggest that the high expression of these 3 DEGs in tumor may be related to DNA methylation, mRNA methylation modification and potential transcription factor regulation. These studies are only preliminary data analysis results and still need further experimental verification. However, these results expand our understanding of the expression regulation of these three prognosis-related genes.

We constructed diagnostic and prognostic models based on mRNA expression data and DNA methylation data of 3 DEGs. We found that the diagnostic efficiency was 92%, while the diagnostic efficiency in our patient cohort was 100%. P-values of Hosmer-Lemeshow test are greater than 0.05 (0.61 and 0.08, respectively), indicating the stability of the diagnostic model. In the prognostic model, the prognosis of high-risk patients is worse. In order to combine clinical information and simplify evaluation conditions, we used nomograms to show the results of multivariate Cox regression and scored them. The calibration diagram verifies that our nomogram has a good predictive effect on the prognosis of patients. However, due to our short follow-up time, further verification cannot be carried out. The expression of these 3 DEGs is closely related to DNA methylation, we used the DNMIVD to construct the methylation diagnosis and prognostic model (23). It was found that the diagnostic efficiency was lower than that of the gene expression based diagnostic model. The prognosis of high-risk patients is also poor. Due to the lack of validation set, we were unable to conduct further evaluation. However, these results suggest that gene expression and DNA methylation may play an important role in the diagnosis and prognosis of PTC. It provides part of the basis for potential clinical translation in the next study.

We evaluated tumor- and immune-related characteristics of different risk groups in the transcriptome-level prognostic model. We found that patients in the high-risk group were associated with older age and more invasive pathological types. The level of proliferation, TMB, SNV neoantigens, aneuploidy scores, and HRD score increased significantly in the high-risk group. This implies the complex heterogeneity of high-risk groups and provides potential epitopes for immune cells to recognize tumor cells (45).The level of immune infiltration and the diversity of TCR increased significantly in the high-risk group, while CD8^+^ T cell decreased. This indicates that T cell function is impaired and tumor immune escape occurred (46), since survival rates of PTC patients increased when CD8^+^ T lymphocytes were infiltrated (47). Continuous antigen recognition in the tumor microenvironment is an important driver of T cell dysfunction (48). Since the function of macrophages in tumors is closely related to their subtypes, we cannot determine which macrophage subtypes are elevated in the high-risk group. These clinical and molecular characteristics suggest the rationality of the high-risk group. TIMER and EPIC algorithms were inconsistent in inferring the infiltration level of other immune cell types, thus we focused on the infiltration level of CD8^+^ T cell. We did not analyze and discuss other immune cells. Due to the most PTCs belonging to C3 immune subtype (inflammatory), there is no significant difference in C1 (wound healing), C2 (IFN-γ dominant), and C6 (TGF-β dominant) between the two groups (31). Therefore, we infer that this 3-gene panel may be a good prognostic model. However, more experimental evidence is needed to support the further mechanism of tumorigenesis and development and the type of immune infiltration.

There are also some limitations in this study. Firstly, the number of scRNA-seq samples is limited. In addition, our analysis is based on the filtered data set, and we cannot handle the real batch effect. Most of the data we obtained are relatively quantitative data, which only explains the tendency of some phenomena and is not absolutely accurate. Due to various factors, the samples we collected also have heterogeneity, but these are also the characteristics of clinical samples. Finally, we only analyzed the gene expression level without paying attention to point mutations, indels, gene fusions, copy number alternations, which will be the content to be studied in the future.

## Conclusions

We used single-cell RNA-seq to focus on the gene changes between PTC tumor cells and normal epithelial cells, screened 3 DEGs related to prognosis, and analyzed that their expression may be related to methylation and transcription factors and tumor immune infiltration. The diagnostic and prognostic models constructed by the 3-gene panel have good effect. It may be a supplementary indicator of traditional prediction methods.

## Data Availability Statement

The original contributions presented in the study are included in the article/**Supplementary Material**. Further inquiries can be directed to the corresponding author/s.

## Ethics Statement

The study protocol was approved by the ethics committee of the General Hospital of Tianjin Medical University (IRB2021-KY-022). The patients/participants provided their written informed consent to participate in this study.

## Author Contributions

XH-H and K-Z designed the study and revised the manuscript. ZY-C collected and analyzed the single cell RNA sequencing and the TCGA-THCA data, and wrote the manuscript. YZ-W collected and analyzed the bulk RNA-seq data of our cohort. DY-L, YT-L and Y-H performed the qPCR experiment. LN-J and CG-Y performed the visualization operation of correlation analysis. ZG-T, WB-S, and FX-L provided the samples and clinical information of patients. All authors listed have made a substantial, direct, and intellectual contribution to the work and approved it for publication.

## Funding

This work was funded by the National Natural Science Foundation of China (NSFC, No. 81672641), the Tianjin Education Commission’s Science and Technology Project (2020KJ153), and Tianjin key Medical Discipline (Specialty) Construction Project.

## Conflict of Interest

The authors declare that the research was conducted in the absence of any commercial or financial relationships that could be construed as a potential conflict of interest.

## Publisher’s Note

All claims expressed in this article are solely those of the authors and do not necessarily represent those of their affiliated organizations, or those of the publisher, the editors and the reviewers. Any product that may be evaluated in this article, or claim that may be made by its manufacturer, is not guaranteed or endorsed by the publisher.

## Supplementary Material

Supplementary Figure 1 Annotation of cell type. **(A)** Marker genes of different cell clusters in 4 tumor samples. **(B)** UMAP of cell clustering in 4 tumor samples. **(C)** Marker genes of different cell clusters in 4 normal samples. **(D)** UMAP of cell clustering in 4 NORMAL samples.

Supplementary Figure 2 Annotation of cell type. **(A)** KM plot of genome-level altered group and unchanged group. **(B-D)** Relationship between DNA methylation sites and gene expression of three prognosis related genes.
