# Supplementary figures and images for: Single-Cell RNA Sequencing Revealed a 3-Gene Panel Predicted the Diagnosis and Prognosis of Thyroid Papillary Carcinoma and Associated With Tumor Immune Microenvironment
Source: Front Oncol. 2022 Mar 11;12:862313. doi: 10.3389/fonc.2022.862313 (PMC8962641; doi:10.3389/fonc.2022.862313)

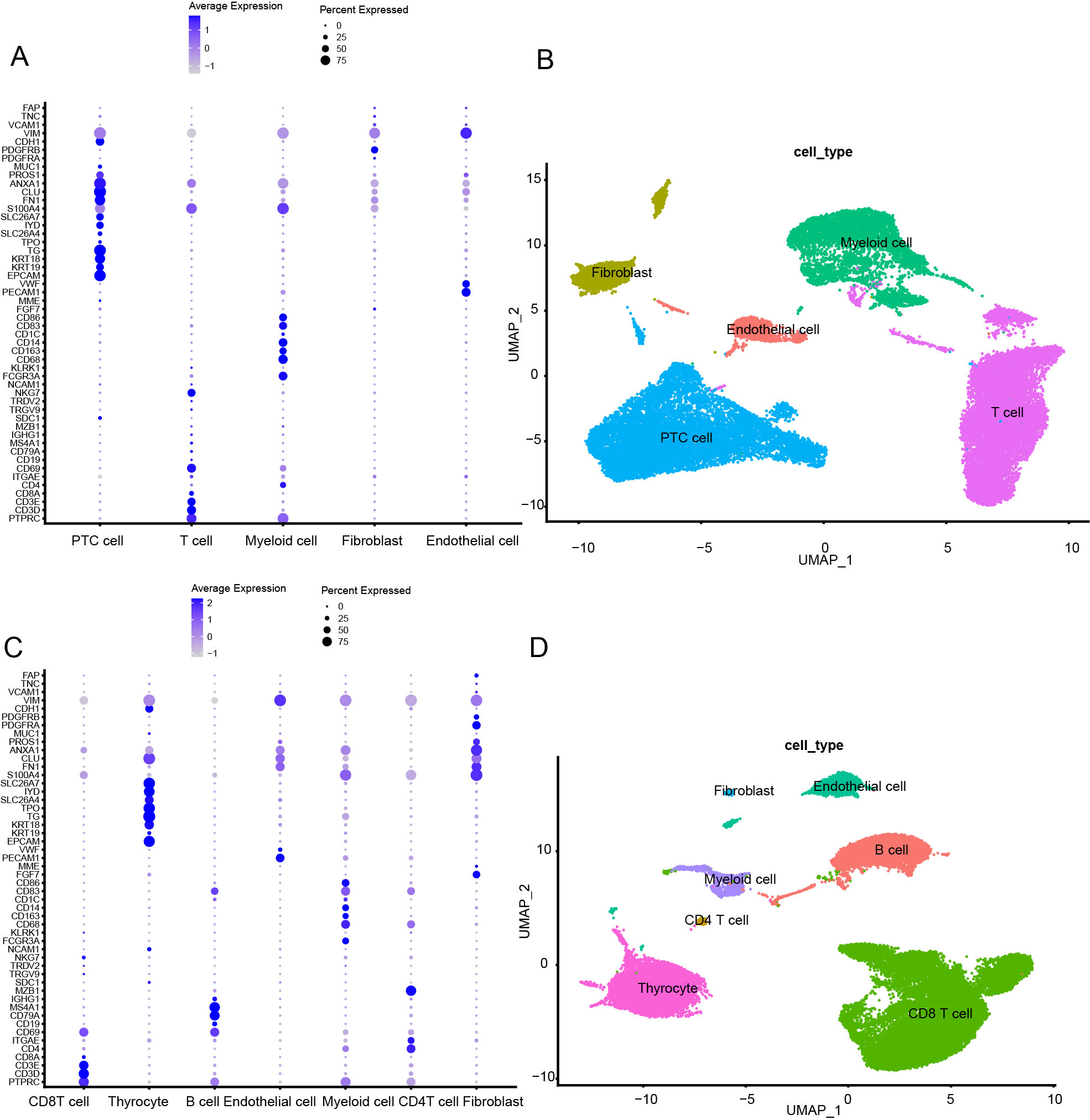

Supplement: Supplementary Figure 1 — Annotation of cell type. (A) Marker genes of different cell clusters in 4 tumor samples. (B) UMAP of cell clustering in 4 tumor samples. (C) Marker genes of different cell clusters in 4 normal samples. (D) UMAP of cell clustering in 4 NORMAL samples. [file Image_1.tif]

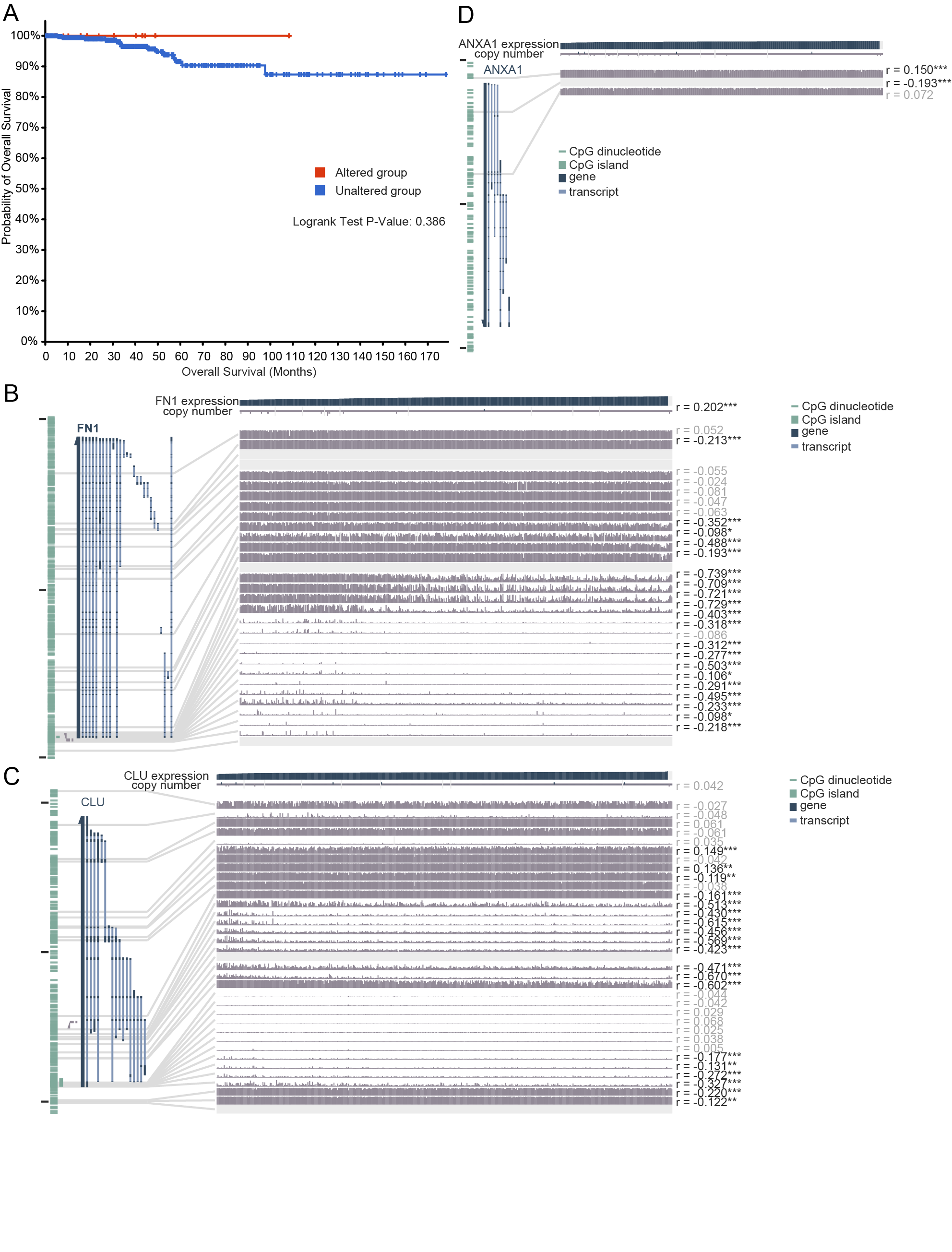

Supplement: Supplementary Figure 2 — Annotation of cell type. (A) KM plot of genome-level altered group and unchanged group. (B-D) Relationship between DNA methylation sites and gene expression of three prognosis related genes. [file Image_2.tif]
